# Supplementary material for: Are carbon emissions trading and green financial instruments synergistic? -Comprehensive quantitative research based on content analysis
Source: PLoS One. 2024 Mar 7;19(3):e0298601. doi: 10.1371/journal.pone.0298601 (PMC10919601; doi:10.1371/journal.pone.0298601)
Supplement: S1 Data — (ZIP) [file pone.0298601.s001.zip › Data/地级市Z_F工作报告.zip!/║■▒▒/╛ú├┼╩╨/└┤╫╘╣1⁄2╓┌║┼í╛┬φ┐╦╩2╛▌═°í┐_2008.docx]

各位代表：

　　现在，我代表市人民政府向大会报告工作，请予审议，并请各位市政协委员和列席人员提出意见。

　　一、2008年工作回顾

　　2008年是极不平凡的一年。面对历史罕见的雨雪冰冻等严重自然灾害和国内外经济环境的重大变化，全市上下坚持以邓小平理论和“三个代表”重要思想为指导，深入贯彻落实科学发展观，按照中央、省和市委的决策部署，团结拼搏，扎实工作，全市经济实现平稳较快增长，各项事业全面进步，社会大局保持稳定。初步核算，全年实现地区生产总值520.4亿元，同比增长14.0%；全社会固定资产投资210.1亿元，增长37.1%；社会消费品零售总额188.9亿元，增长24.6%；进出口总额3.27亿美元，其中出口2.09亿美元，增长20.7%；实际利用外资1.19亿美元，增长15.6%；完成地方财政总收入34.8亿元，增长18.6%，其中地方一般预算收入14.7亿元，增长21.8%；城镇居民人均可支配收入12690元，增长14.6%；农民人均纯收入5332元，增长14.6%；居民消费价格指数上涨5.8%；城镇登记失业率为4%；人口自然增长率为2.97％。；单位生产总值能耗下降5.2%，化学需氧量、二氧化硫排放量分别下降3%和2.4%。除居民消费价格指数外，市七届人大二次会议确定的主要预期目标均已完成或超额完成。

　　一年来，我们主要做了以下工作：

　　（一）积极应对宏观环境变化，保持经济平稳较快发展。在年初抗击雨雪冰冻和随后多次局部暴雨洪涝等严重自然灾害中，全市上下坚持一手抓抗灾救灾，一手抓经济社会发展，在大灾之年实现平稳开局，取得了好于灾后预期的发展成绩。针对生产要素供给偏紧、能源和原材料价格暴涨以及部分企业效益下滑等巨大困难，千方百计抓发展、保稳定，加强煤电油运等生产要素组织调度，强化金融服务支持，努力保障经济正常运行，做到了各项工作统筹兼顾，有序推进。面对金融危机影响逐步加深的态势，在落实中央、省扩大内需政策措施的同时，出台了扶持企业经营发展、创建投资项目绿色通道等举措，着力解决制约发展的突出困难和问题，促进了经济发展。

　　（二）全力推进项目建设，培植发展后劲。坚持“大抓项目、抓大项目”，开展“项目建设年”活动，建设了一批重点项目。全年施工项目1027个，增加119个，其中亿元以上项目99个，增加64个。天茂集团年产100万吨二甲醚二期、众和纺织20万锭紧密纺一期、葛洲坝水泥厂子陵日产4800吨水泥熟料、鄂中化工年产20万吨复合肥、洋丰中磷120万吨采选矿、楚玉食品一期工程、福耀汽车玻璃等项目竣工投产，李宁（荆门）工业园、中集(宏图)能源装备生产基地、凯迪生物质能发电、汇源集团钟祥果蔬饮料食品加工等项目开工建设。抢抓国家扩大内需的机遇，全年争取国家和省专项资金5.7亿元，储备项目1200多个，为经济社会发展增添了后劲。

　　（三）调整产业结构，转变发展方式。扎实推进“百亿工程”，着力打造“300亿石化，100亿磷化、建材、食品产业”，四大重点产业规模以上企业发展到516家，资产总额、实现主营业务收入均超过全市规模工业总量的三分之二。规模以上工业实现增加值172亿元，增长25%。加快高新技术产业发展，高新技术产业产值突破100亿元。京山、钟祥、沙洋经济开发区发展势头强劲，工业重镇建设方兴未艾，207国道工业走廊成为全市工业经济新的增长点。积极培育壮大市场主体，全市规模以上工业企业达784家，比上年末净增170家。东光集团、中天集团、荆襄集团改革不断深化，国企改制遗留问题正在逐步解决，上市后备企业培育工作稳步推进。启动国家循环经济试点市建设，试点方案顺利通过国家发改委评审，节能减排和循环经济发展取得新的进展。全年共实施44个重点循环经济项目，完成投资25.3亿元，其中26个项目获国家9100万元的专项资金支持。文化旅游业等服务业成为新的经济亮点。客店镇被列入全省“十大旅游名镇”实验区。明显陵、黄仙洞被评为国家4A级景区。成功举办油菜花旅游节、鄂西生态文化旅游圈荆门推广周和中原万人游荆门等活动，全年共接待国内外游客544.4万人次，实现旅游业总收入21.9亿元，分别增长7.1%和17.2%。荆门被评为“中国特色魅力城市”。现代物流、金融、保险等服务业加快发展，服务业实现增加值增长11.8%。全市金融机构各项存款余额425.5亿元，比年初净增81亿元，增长23.5%；各项贷款余额206.4亿元，按可比口径比年初净增19.2亿元，增长10.3%。

　　（四）加强“三农”工作，统筹城乡发展。坚持把促进农民增收作为首要任务，努力克服自然灾害和农资涨价、农业比较效益下滑等不利因素影响，农业在大灾之年获得丰收，提前两年完成了“十一五”规划主要指标。主要农产品产量稳定增长，全年粮食总产240.5万吨，增长7.2%；油料总产34.75万吨，增长39.6%；生猪、家禽、肉类、禽蛋、水产品产量保持大幅增长。农业产业化经营深入推进，建成各类畜禽养殖小区120个、万头猪场13个，新增国家级龙头企业1家，新增年销售收入过亿元的龙头企业10家、过5亿元的5家、过10亿元的1家，产业化龙头企业销售收入突破100亿元。农村专业合作组织发展较快，新增各类专业合作组织107个，达到343个。国家现代林业示范市、全国农机化示范区、全国节水型社会建设试点市建设取得新成效。全面落实各项强农惠农政策，全年各项惠农资金达到4.5亿元。加强农村基础设施建设，解决了15.04万人的饮水安全问题，建设农村户用沼气池2.18万口；新建通村公路523公里、通组公路路基5140公里、碎石路面2018公里；完成了4个乡镇、55个行政村的新农村电气化建设任务。

　　（五）努力改善民生，促进社会和谐。深入开展“察民情、排民忧、解民难”活动，解决了一些关系群众切身利益的问题，市政府承诺的十件实事全面完成。一是在城市建设方面，投资1.24亿元，在中心城区实施了一批道路、园林绿化和公共公用设施项目，配套完善了荆门经济开发区的部分市政基础设施。拆除中心城区违法建筑万余平方米。开展中心城区粉尘、烟尘、废气等污染治理，中心城区环境空气质量达到国家二级标准以上天数占88.8%。加快交通基础设施建设，武荆高速公路荆门段建设稳步推进，国道、省道改造工程顺利实施。二是在就业和社会保障方面，扶持1413人创业，新增就业3.57万人，转移农村劳动力4.7万人。积极推进城镇居民基本医疗保险试点工作，参保居民44.4万人，占应参保面的81%。全面实施新型农村合作医疗制度，参合率达90.8%。农村社会养老保险正式启动。经济适用住房新开工32.25万平方米，竣工23.95万平方米，对城镇人均住房面积10平方米以下的4804户家庭发放廉租住房补贴488.7万元；完成1278户农村特困户危房改造任务。三是在教育、文化、体育和卫生方面，全部免除了我市城乡25万名义务教育阶段学生学杂费，按每生1500元标准资助2.1万名贫困中职学生入学，按每生3000元标准资助200名大学贫困新生入学。荆门职教集团顺利组建，儿童育教大楼如期建成。建成33个村级文化活动中心、9个文化中心户、93个农民健身工程项目和503家农家书屋，《十二月等郎》应邀在国家大剧院成功展演，并在上海等地进行了商演。以迎奥运为主题的群众性体育活动蓬勃开展。荆门籍运动员在北京残奥会上取得4金2银2铜的优异成绩。省十三运会筹备工作全面启动。广播电视事业发展取得新突破，城区数字电视整体转换达到8万余户。公共卫生体系建设得到加强，全市疾病预防控制和突发公共卫生事件应急能力进一步提高，手足口病得到有效防控，完成了100名晚期血吸虫病人的免费救治任务。人口和计划生育工作取得新进展。四是在社会建设和管理方面，深入开展食品、矿山、危化品安全隐患的排查和专项整治，各类事故得到有效遏制。认真做好群众来信来访工作，进一步规范信访秩序。深入开展“五五”普法活动，法治荆门建设取得新成效。实施公安“三基”工程，社会治安防控体系不断完善，综合治理工作进一步加强。军队转业干部、复员退伍军人接收安置和优抚工作得到落实。驻荆部队和预备役人员积极参加抢险救灾等急难险重任务，为维护社会稳定作出了积极贡献。审计、统计、物价、工商、民族、宗教、烟草专卖、对台、外事、侨务、气象、民兵预备役、人防、防震减灾、档案、地方志、保密和工会、青年、妇女、儿童、老龄、残疾人、应急管理、住房公积金管理等各项工作都取得新成绩。

　　（六）加强效能建设，提高政府执行力。深入开展“解放思想、开放崛起”大讨论，组织实施项目建设、安全生产、城乡环境综合整治“三个百日大会战”和提高政府执行力、加强文明执法教育“两项活动”，政府及其工作部门效能和机关作风进一步改善。推进依法行政，自觉接受市人大及其常委会法律监督、工作监督和市政协民主监督，坚持重大事项向市人大及其常委会报告和向市政协通报制度，办理人大议案、代表建议、政协提案449件。深入贯彻实施《行政许可法》，建立行政审批首席代表制，试行并联审批，行政许可和审批事项进一步削减。推进政府信息公开，加强电子政务建设。坚持不懈地开展反腐败斗争，纠正行业不正之风和治理商业贿赂工作取得了新成效。

　　各位代表！抗震救灾是一项严肃的政治任务。面对汶川特大地震灾害，全市上下发扬“一方有难、八方支援”的优良传统，累计捐款3009万元，捐物折款82.5万元。根据省对口援建要求，我市及时成立援建领导小组和工作专班，迅速进驻四川省汉源县开展工作，该县万里110kV变电所项目和市荣乡中心小学、卫生院项目均已开工建设。与此同时，援建新疆温泉县工作进展顺利。

　　各位代表！过去的一年，我市经济社会发展取得的成绩和进步来之不易，这是省委、省政府和市委正确领导、科学决策的结果，是全市人民不畏艰难、团结奋斗的结果。在此，我谨代表市人民政府，向全市人民，向给予政府工作大力支持和有效监督的人大代表、政协委员和各民主党派、工商联、无党派人士、各人民团体，向中国人民解放军驻荆部队、武警部队，向关心支持荆门建设发展的中省在荆单位和各界人士，表示崇高的敬意和衷心的感谢！

　　在看到成绩的同时，我们也清醒地认识到，经济社会发展和政府工作中还存在不少问题。主要是：受金融危机的影响，部分企业生产经营困难；长期形成的结构性矛盾和粗放型增长方式尚未有效改变，资源环境制约更加明显，节能减排任务艰巨；农业基础脆弱，农民持续增收难度较大，农村生产生活条件亟需进一步改善；财税形势不容乐观，收支矛盾更加尖锐；就业、社会保障、物价、住房等关系群众切身利益的问题需要继续下大力解决，维护社会稳定的任务仍然繁重；政府自身改革和建设有待进一步加强，一些部门办事效率不高，部分政府工作人员服务意识和依法行政观念不强、作风不实，等等。对此，我们要进一步增强忧患意识和责任感，更加兢兢业业、扎扎实实地做好政府各项工作。

　　二、2009年的形势和任务

　　今年是建国60周年，是实施“十一五”规划的关键之年，也是我们以特别之为迎接特别挑战、推动科学发展上水平的拼搏奋斗之年。做好今年的工作，具有极其重要的意义。

　　当前，我市经济社会发展的外部环境日趋复杂，形势严峻。国际金融危机尚未见底，对实体经济的冲击和影响逐步加深。外部需求明显减弱，我国经济运行中的困难增加，经济下行压力加大。我市经济在国际、国内环境的双重挤压下，竞争压力增大，资源环境约束加剧，产业结构矛盾凸显，保投资增长、保企业发展、保财政平衡、保城乡居民增收和就业、保社会稳定的难度很大，今年可能是近些年来荆门经济社会发展最困难的一年。我们必须充分估计，勇敢面对，切不可掉以轻心。

　　尽管挑战空前严峻，但辩证思维，我们面临的机遇和条件也前所未有。一是国家出台了扩大内需、促进经济增长的一系列政策措施、作出了推进农村改革发展的一系列重大部署并深入实施促进中部地区崛起战略，省委、省政府全面启动了鄂西生态文化旅游圈建设，以及这次金融危机形成的倒逼机制，等等，都有利于我们进一步放大“国家循环经济试点市”、“中国特色魅力城市”等金字招牌效应，积极有效地抓招商、争项目、调结构、扩内需、保增长。二是我市内生型经济结构特征明显，主导产业和工农业产品与国家扩大内需的产业方向基本一致，受国际经济波动影响的程度相对较小。特别是近两年来，我市经济发展势头较好，一批大集团、大项目相继落户，重点骨干企业不断发展壮大，规模企业显著增加，新增固定资产投资效应正在逐步显现，发展后劲明显增强。三是我市有一批优秀的中省企业家、本土企业家和外来企业家，他们思进取、谋发展、图崛起的积极性很高，支撑经济较快发展的动力依然强劲。四是我市承办省十三运会，一批城市基础设施项目正在或即将开工建设，为我们进一步提升城市品位和促进城市经济发展提供了难得的契机。

　　黄金有价，信心无价。抓住一次机遇，就能赢得一次跨越。我们坚信，有省委、省政府和市委的正确领导，有市人大、市政协的监督和支持，有学习实践科学发展观形成的强大动力，有历届市委、市政府“打基础、管长远”积蓄的发展能量，有全市人民的奋力拼搏，我们一定能够化挑战为机遇、变压力为动力，牢牢把握科学发展的主动权，努力“在爬坡中提速，在转弯处赶超”，继续保持经济平稳较快发展的良好势头。

　　今年政府工作的指导思想是：认真贯彻党的十七大、十七届三中全会、中央经济工作会议和省委九届五次全会精神，按照市委六届六次全会部署，以科学发展观统领经济社会发展全局，紧紧围绕“保增长、促发展”的总体目标，着力推进改革、开放、创新，着力推进产业优化升级和发展方式转变，着力改善民生和维护社会稳定，推动经济社会又好又快发展，加快工业强市、现代农业强市、文化旅游强市和生态宜居城市建设步伐。经济社会发展的主要预期目标是：全市地区生产总值增长12%，单位生产总值能耗下降4.8%，化学需氧量和二氧化硫排放量分别下降1.5%、0.5%，全社会固定资产投资增长33%，规模以上工业增加值增长20%，社会消费品零售总额增长20%，外贸出口和直接利用外资分别增长12%，地方一般预算收入增长13.5%，城镇居民人均可支配收入和农民人均纯收入分别增长10%和8%，居民消费价格涨幅控制在4%左右，城镇登记失业率控制在4.5%以内，人口自然增长率控制在4％。以内。围绕以上目标任务，今年我们将着力抓好以下重点工作。

　　（一）坚持以扩大投资为引擎，全力保增长

　　充分调动政府、企业、银行和社会的积极性，积极向上争取中央和省投资、向外引进产业投资、向内激活民间投资，以扩大投资力促发展。

　　坚定不移地大抓项目、抓大项目。围绕循环经济发展、新农村建设、鄂西生态文化旅游圈建设、城乡基础设施建设和改善民生，积极向上争取项目。力争全年续建和新开工亿元以上的项目不少于170个，计划投资不少于200亿元，其中5亿元以上项目34个，计划投资近100亿元。加快重点项目建设，尽快开工建设大峪口中海油化肥基地、华能2&times;30万千瓦热电联产、10万吨燃料乙醇和葛洲坝京山日产4800吨新型干法水泥等项目，加快李宁（荆门）工业园、中集(宏图)能源装备生产基地、天茂集团新能源基地和宝源木业工业园等项目建设步伐，确保洋丰30万吨磷酸二铵、汇源集团钟祥果蔬饮料食品加工、金龙非织造布扩建、楚玉食品二期和京山光达石材加工等项目建成投产。做好京山伟嘉工业园、沙洋林纸一体化、引江济汉工程沙洋段、钟祥碾盘山水利枢纽工程及核电等项目的前期工作，力争尽快落地。对项目建设和扩大投资工作成绩突出的单位给予重奖。

　　加大招商引资力度。坚持大员招商、全民招商、产业招商，理顺招商体制，优化招商环境。只要符合科学发展观和环保政策，不论重工业还是轻工业，不论高新技术产业还是中低产业，不论规模大、中、小，都欢迎在荆门投资落户。强化招商引资责任，坚持实行“二分之一工作法”和小分队专班招商等办法，精心组织参加中博会、鄂港粤经贸洽谈会、厦洽会、高交会、东北亚博览会等重要经贸活动，拓宽招商渠道，确保完成招商引资100亿元以上的目标。

　　进一步激活民间投资。认真落实推动全民创业的若干意见，完善扶持创业的多方合作联动机制，放宽条件，降低门槛，优化环境，激发全民投资创业的激情和潜能，营造“投资创业有功、发展民营光荣”的良好氛围。完善鼓励民间投资的信贷政策，支持信用担保机构为民间投资和民营企业贷款提供担保，拓宽民间投资的融资渠道。引导民营企业加大投入、扩大产能、强化技改、加强管理，努力提高信用等级和市场竞争力，实现“二次创业”。力争今年新增民营企业500家，启动民间资本新增50亿元。

　　加大财税金融支持经济发展的力度。加强财源建设，努力做大财政“蛋糕”。全面落实国家、省支持经济增长的财税政策，发挥财政资金的政策引导作用，市财政筹措7000万元支持重点企业发展和重点项目建设。鼓励金融机构加强和创新信贷服务，确保全年新增贷款不低于30亿元。加强银政银企合作，推动国有商业银行省分行与市政府签署金融合作协议。组织开展“政府支持银行、银行支持企业”和“行长企业行”活动，促进市内金融系统加大信贷投入。积极引进区域性股份制商业银行，创造条件组建地方性商业银行。组建1家村镇银行和2家小额贷款公司，探索建立创业担保机构，完善信用担保体系。推进政府资产资本化，积极引进风险投资，支持有条件的企业发行债券。加强金融监管，优化金融生态环境。规范保险经营行为，促进保险业稳步发展。

　　（二）着力推进企业振兴和产业升级，提高工业经济运行质量和效益

　　把发展循环经济作为保增长、促发展的着力点，加快调整产业结构，转变经济发展方式，确保工业增长。

　　全力服务企业发展。牢固树立“产业第一、尊重企业家”的理念，认真落实中央、省、市扶持企业经营发展的一系列政策措施，支持重点企业加快发展，扶持潜力企业尽快成长，帮助困难企业尽快走出困境。扎实开展“服务企业（项目）年”活动，实行市级领导包联和工作专班服务重点项目、政府部门对口帮扶重点企业、企业点名服务等制度，充分发挥中小企业服务中心（工业110）的功能，建立市、县（市、区）服务企业“直通车”制度。抓好煤电油运、原材料供应等生产要素组织协调，加强预警监测，确保重点行业和企业满负荷生产。支持引导企业适应市场变化，大力开拓市场。积极帮助企业解决用地、用工等难题，切实减轻企业负担。

　　大力推动产业优化升级。一是培植优势产业集群。实施“15311”工程，围绕打造1000亿化工、500亿食品、300亿建材、100亿机电、100亿轻纺等产业的发展目标，优化产业结构，力争二、三产业在三次产业结构中的比重提高1个百分点。突破性发展电子信息、新医药、新材料、新能源等高新技术产业，力争高新技术产业完成增加值占规模以上工业增加值的比重达到20%。实施“项目满园工程”，力争荆门经济开发区和各县（市、区）工业园区进入全省先进开发区（工业园区）行列。加快推进工业项目向沿线集中区和工业重镇集中，打造独具特色的207国道工业走廊。二是优化企业结构。按照做大做强的方向，大力推进企业兼并重组，鼓励优势企业强强联合，组建大企业大集团，培育行业龙头。支持洋丰、葛洲坝水泥、京兰水泥等龙头企业兼并生产规模小、生产工艺落后的中小企业，支持关联企业、上下游企业联合重组，实现一体化经营，规避市场风险。鼓励企业向专、新、特、精方向发展，提高配套协作水平与市场竞争力。深入实施“中小企业成长工程”，力争全市规模工业企业总数达到1000家。三是培育精品名牌。引导企业根据国内外市场变化和客户需求，自主开发新产品、新技术，延伸产业链条。支持企业采用先进技术、先进生产工艺和设备，提高产品质量。坚持质量兴市战略，加强对金龙泉啤酒、洋丰磷肥、国宝桥米等名牌、名品的扶持和自主知识产权的保护，认真落实驰名商标和名牌产品奖励政策，力争再创1至2个中国驰名商标。

　　提升循环经济发展水平。坚持把项目建设作为推进循环经济发展的重要抓手，全面落实循环经济试点实施方案，今年组织实施循环经济重点项目160个，完成投资110亿元以上。抓紧开工建设葛洲坝水泥厂余热发电、雄韬电源公司太阳能（风能）储能电池生产、金龙泉综合节能改造及副产品综合利用、立业生物科技循环经济园等一批循环经济项目。推动天茂集团节水改造、格林美含锡废弃物综合利用、凯迪电力生物质能发电等一批重点循环经济项目尽快建成投产。继续开展循环经济进企业、进机关、进学校、进家庭活动，积极创建零排放车间、清洁生产班组和节约型机关（学校、家庭）。按照“硬标准、硬约束、硬检验”的要求，强化节能减排工作责任制，坚决遏制高耗能、高排放行业增长。开展全民节能行动，在全社会推行节能、节电、节水等措施，抓好重点行业、重点领域的节能减排，全面实施低效燃煤锅炉改造、区域热电联产、绿色照明等节能工程，继续开展“九小”专项治理。

　　（三）突出增产增收，奋力推进现代农业强市建设

　　全面贯彻党的十七届三中全会精神，坚持农业产业化、农村城镇化、农业机械化、服务社会化“四位一体”，扎实推进农村改革发展，力争我市现代农业发展和社会主义新农村建设走在全省前列。

　　大力发展现代农业。一是调整农业结构。围绕规模化、市场化和标准化，高标准建设好200万亩优质稻和10万亩有机稻、200万亩“双低”油菜、50万亩优质棉花、400万头生猪和4000万只家禽、100万亩特色水产、185万亩速生丰产林和100万亩高效经济林等优势农产品基地，抓好京山食品加工园、沙洋农业循环经济示范园、钟祥台湾农民创业园、东宝生态农业园、掇刀花卉苗木科技园、屈家岭现代农业示范区等农业园区建设，力争新认证“三品”标识产品50个，农产品标准化生产达到80%，进一步巩固和提升粮食、油料、畜禽、水产、林果、棉花和生物质能源七大产业。二是创新农业产业化经营模式。落实各项扶持政策，培植钟祥雨润、楚玉食品、湖北爱斯曼等一批带动力强的龙头企业，引导国宝桥米、中粮祥瑞、宝源木业等企业进行并购重组、品牌整合，发展精深加工，重点实施湖北“一袋米”工程第三步发展战略和湖北“一壶油”工程，大力推进桥米百亿产业链建设，力争新增年销售收入过亿元企业10家，钟祥、京山农产品加工产值分别达到100亿元左右，其他县、区分别达到50亿元左右。三是创新农业社会化和科技服务体系。积极发展和规范各类农民专业合作组织，进一步拓展功能，力争新增各类农民专业合作组织120个。着力推进农业科技创新，重点支持种子工程、丰产栽培、农业节水、疫病防控、防灾减灾等领域的科技创新和推广。

　　加强农村基础设施建设。发挥“国家现代林业示范市”、“全国节水型社会建设试点市”、“全国农机化示范区”等品牌效应，争取中央、省级投资，加快农村基础设施建设。加强农业综合开发，切实改造中低产田、低丘岗地、低产林地，整乡、整村推进土地整理和复垦。加快农村水利建设步伐，抓好以汉江河道堤防综合治理和37座病险水库除险加固为重点的防洪工程，以漳河、温峡、惠亭、石门等灌区续建为重点的灌溉工程，以改善生态环境为重点的水土保持工程，以小型农田水利建设为重点的高产农田建设工程，完善防汛抗旱工程体系。加快农村路网、电网、广播电视网等建设，逐步推进“因特网”进农村。

　　拓宽农民增收渠道。积极拓展农业功能，鼓励农民发展集约经营和精品农业、生态农业、观光农业、休闲农业和农庄经济，依靠资源优势发展特色食品加工、工艺品制作等产业，不断增加收入。抓好农超对接及各类展销，推进农产品进城。实施“农村经济状元培育工程”，培植农村经济能人。通过盘活存量资产、改造升级现有企业、实施中介服务等途径，加快农村集体经济发展。坚持整村推进和开发式扶贫方针，做好新一轮扶贫开发基础性工作，支持革命老区、库区和少数民族地区加快发展。扎实开展南水北调中线工程丹江口库区移民试点工作。

　　切实抓好农村各项改革和政策落实。按照依法、自愿、有偿原则，逐步建立农村土地承包经营权和林地流转市场，有序流转土地（林地）承包经营权，探索发展多种形式的土地适度规模经营。鼓励有条件的地方发展专业大户、家庭农场、农民专业合作社等规模经营主体。全面完成集体林权制度改革。多渠道增加农业投入，加大项目争取和资金整合力度，逐步增加地方财政对农业的投入，积极支持、引导社会资本参与农业农村开发。支持邮政储蓄银行开办农村土地承包经营权证质押贷款试点。以县为单位，组建农业担保公司，探索建立农民合作社授信制度。按照依法依规、民主公开原则，在东宝区开展化解村级债务工作试点。加快推进城中村、城郊村和园中村集体资产产权制度创新试点，探索集体资产的有效实现形式。落实国家增加农业补贴、扩大补贴范围等一系列强农惠农政策，完善补贴方式，实行粮食直补、良种补贴、农机具购置补贴、农资综合直补、林业生态效益补偿“一折通”，确保农民直接受益。认真落实重点粮食品种最低收购价政策，促进农产品价格保持在合理水平。控制农业生产资料价格过快上涨。

　　（四）大力发展现代服务业，增强消费对经济的拉动作用

　　突破性发展文化旅游业。继续实施“143”文化旅游发展战略，努力把荆门打造成全省重要旅游节点城市和目的地，在鄂西生态文化旅游圈中发挥更大作用。一是加快发展中心城区文化旅游业。落实《荆门市中心城区旅游开发总体规划》，组建荆门市旅游集团，加快建设象山风景区、东宝山风景区、漳河风景区、仙居乡村游风景区和荆门特色餐饮一条街、旅游商品一条街，着力打造“宜居、宜商、宜游”的文化旅游名城。二是加快旅游景区建设。整合开发虎爪山、大口、太子山森林公园旅游资源，联动开发大洪山风景名胜区，抓好京山温泉国际旅游度假区、楚国第一城等旅游项目建设。深入开展旅游名镇、名村创建活动，对旅游名镇进行考核奖励。三是加快以交通为重点的旅游基础设施建设。积极服务长荆铁路电气化改造、荆岳铁路建设及荆门火车站改造工程。开工建设随岳高速公路三阳互通至屈家岭的旅游公路，做好荆潜高速等有关新建高速公路前期工作。完成国省道大修60公里、县乡道改造50公里建设目标，确保武荆高速公路荆门段年内建成通车。积极发展星级宾馆和经济型连锁酒店，建设荆门游客咨询服务中心，扶持成立一家国际旅行社，引导规范农家乐升级改造。四是强化旅游宣传促销。办好油菜花旅游节、漳河水域游泳公开赛及荆门人游荆门等节庆活动，力争全年接待国内外游客600万人次，旅游业总收入增长18%。

　　积极发展现代物流业和房地产业。紧密结合工农业的发展，大力发展生产性服务业，重点实施农产品批发市场、农资市场标准化和粮食现代物流设施改造工程，加快李宁公司物流产业园、星球商业中心、人民电器集团物流园、荆乐置业五金机电大市场等项目建设，引进培育第三方物流企业。抓住市场、政策机遇，加强廉租住房等保障性住房建设，着力解决城市低收入家庭住房难和住房差的问题。认真落实住房税收、信贷政策，支持和鼓励改善型住房消费，加快发展二手房市场和住房租赁市场。完善住房公积金制度，调整住房供应结构，重点支持中小户型、中低价位普通商品房开发。支持搬迁企业异地新建职工住房。根据企业务工人员的生活特点，就近开发建设农民工公寓。逐步整治、改造旧住宅区和棚户区。

　　着力拓展城乡消费市场。建立完善工资正常增长机制和支付保障机制，提高城乡低保补助标准、企业退休人员基本养老金水平和优扶对象生活补助标准，增加城乡居民特别是中低收入者收入，提高消费能力，稳定消费预期。进一步拓宽消费领域，稳步扩大住房、汽车等大宗消费，大力发展小区商业、物业、家政等服务性消费，积极开发旅游、文化、体育健身和网络等热点消费，促进城市耐用品消费升级换代，规范并积极发展旧货市场，健康发展信息、中介和社区服务业，促进各种新型业态竞相涌现，满足不同消费群体联动消费。支持东方百货、金城集团、中商集团荆门公司等商贸流通企业进一步做大做强。继续实施“万村千乡市场工程”、“双百市场工程”和“新农村现代流通服务网络工程”，全面推进“家电下乡”，积极拓展农村消费市场。稳定物价，强化监管，搞好消费维权，改善消费环境，让人民群众放心消费。

　　（五）提升城市功能，加快城乡一体化进程

　　加快中心城区基础设施建设。以承办省十三运会为契机，以创建国家园林城市为抓手，大力实施道路骨架、环境综合整治、公共设施配套、园林绿化四大工程，以更大的魄力、更强的力度推进南城区建设。加快建设城市运动公园、凤凰河公园和千佛洞森林公园，高标准建设漳河防洪快车道及来龙、长坂坡、龙井、凤凰、掇刀石等大道。着力推进人力资源市场、动物疫病控制中心、交通信息中心、地产交易中心等大型公共建筑项目建设。完善南城区给排水、电力、电信、燃气等线路管网建设。开工建设城东220千伏输变电工程。狠抓城市绿化、亮化、美化，建成杨湾广场、千佛洞广场，打造象山大道南段绿化景观带，亮化城市主要街道和重要建筑物。配套建设小街小巷、游园、公厕、停车场、公交总站等公用设施。

　　严格城市规划和管理。进一步提高规划水平，加快完成荆门城市总规修改，加大控制性详规的编制力度，完善园林绿地、景观水系、地下管网等专项规划，开展特色街区、城市进出口街景等专项设计，全面提升规划水平。严厉打击违法建设行为，切实扭转城区无序建设局面。以创建国家文明城市、卫生城市、环保模范城市为目标，探索推行网格化模式，逐步实现城市管理的社会化、精细化、信息化，强化“门前三包”和路段管理责任，加强对市容环境卫生、城市垃圾的管理，重点整治中天街等主要街道、菜市场、城乡结合部的经营秩序，着力解决“脏、乱、差”等突出问题，努力创造整洁有序、和谐优美的城市环境。

　　推进城乡经济社会一体化发展。按照“一主三化”的方针，加快县域经济发展，支持各县市区重点发展2-3个带动力强和吸纳农村剩余劳动力成效明显的产业集群，打造区域品牌。学习借鉴仙洪新农村建设试验区和钟祥客店旅游名镇建设经验，加快钟祥、京山、沙洋三个县城和小城镇建设步伐，突出抓好10个工业重镇、10个旅游三产重镇建设，把城镇建成吸纳农村剩余劳动力的“蓄水池”、农民走向市场的“桥头堡”、农副产品流通的“集散地”和经济发展的“主战场”。支持掇刀区从探索土地流转方式、健全社会保障机制、统筹城乡规划建设等方面入手，整区推进城市化，努力创建城乡一体化示范区。

　　加强生态建设和环境保护。深入开展“蓝天、碧水、绿地、宁静”行动，加强漳河生态监察工作，建成全国试点区域。加强中心城区环境监管，增设和改造空气自动监测站，全面实施排污许可证制度，大力推进重点污染源在线监控设施建设，积极化解化工企业环境风险。加大饮用水源的保护力度，严禁水库投肥养殖。加强汉江、长湖等重点河流、重点水源生态环境的修复和治理。加快竹皮河流域水污染综合治理和城镇污水、垃圾处理设施建设。加强农村生态环境建设，以城乡结合部、主要交通干线、工业和旅游三产重镇、新农村建设示范村为重点，以实施生态景观、住宅建设与改造、生态家园、环保清洁等工程为抓手，扎实推进农村环境综合整治。加强“绿色学校”、“绿色社区”和“环境友好企业”创建工作。

　　（六）深化改革开放，增强发展活力和动力

　　创新体制机制。积极稳妥地解决好国有、集体企业改制遗留问题。做好凯龙、格林美、洋丰、宝源等企业上市直接融资的前期准备工作，推进东光引进战略投资者。加快完善商贸、粮食、交通、文化、科技等非工业领域国有企业的产权制度改革。改革预算管理，逐步建立项目评审和绩效评价制度，不断提高财政资金使用效益。完善国库管理体系，提高国库资金直达比例，

　　扩大政府采购范围。完善“乡财县管乡用”制度，规范村级财务收支行为。扎实推进增值税转型、燃油税等税收制度改革。探索乡镇政府职能定位和具体实现形式，完善“以钱养事”机制。加大公用事业单位改革力度，推行政企、政事、事企分开，发展各类行业协会和中介组织。做好农村义务教育“普九”债务化解工作。继续推进行政审批制度改革，完善网上审批和首席代表工作机制，推行并联审批制度，规范招投标工作，提高审批透明度和审批效率，更好地为群众和企业服务。

　　积极承接产业转移。以海纳百川、兼收并蓄、合作共赢的胸怀，积极拓展对外开放的广度和深度。坚持用硬措施改善软环境，营造安商、亲商、富商的浓厚氛围。发挥我市资源和低商务成本的优势，把握世界500强、中国500强、中国民营企业500强及港澳台等地重点企业的投资动向，积极承接国际资本和沿海产业“双转移”。主动对接武汉城市圈，加强与协作区城市的互惠合作，支持京山作为观察员在武汉城市圈中发挥更大的作用。加快“大通关”进程，支持有实力的企业“走出去”，开拓国内外市场。用足用活中央、省外贸发展和国际市场开拓资金、出口退税等优惠政策，进一步壮大外贸出口主体，重点扶持天茂集团、中集（宏图）能源装备、普安阀门、钟祥金汉江、沙洋楚玉食品等有潜力的出口企业加快发展，努力提高化工医药、机电产品和农产品的出口份额。

　　（七）切实改善和保障民生，促进社会事业全面发展

　　千方百计扩大就业。全面实施创业带动就业计划，做好大中专毕业生、返乡农民工、城镇“零就业家庭”、军转干部和复退军人等群体的就业工作。落实优惠政策，引导企业特别是国有控股、参股企业尽量不裁员或少裁员。做好“迎接新市民”工作，强化技能培训和职业中介、岗位对接等就业服务，为返乡农民工创业、就业提供服务平台。加强大学生就业指导和服务，鼓励和支持他们自主创业。以创建“充分就业社区”活动为载体，开发更多的公益性就业岗位，开展多层次的就业推介会，帮助就业困难人员特别是零就业家庭人员实现就业。加强劳动力市场建设，完善就业服务体系，构建统筹城乡劳动力就业的平台。

　　加强社会保障体系建设。着力完善惠及城乡的社会保障体系，确保全年社会保险扩面新增3.5万人、征收社会保险费11亿元。努力扩大养老保险覆盖面，落实5.86万名企业离退休人员养老金调整政策，逐步将农业“小三场”职工纳入养老保险范围。探索计划生育家庭户、村干部、乡村医生等群体参加养老保险办法。认真落实被征地农民社会保障政策，做好钟祥新型农村社会养老保险试点工作。贯彻国务院《关于深化医药卫生体制改革的意见》，加强医疗保障体系建设，完善城镇职工、城镇居民和农村新型合作医疗制度，将城镇居民医疗保险住院报销比例整体提高10%，年度统筹基金最高支付限额提高到15万元；将新农合基金筹资额度提高到100元/人。做好工伤、生育和失业保险工作。健全城乡社会救助体系，抓好农村低保扩面，提高农村五保户和农村分散供养孤儿的供养标准。改造农村特困户危房1500户以上。

　　加快发展社会各项事业。深入推进义务教育经费保障机制改革，提高基础教育质量。以荆门职教集团为依托，进一步扩大中职学校招生规模，加快培养一批适应产业发展的中高级人才和熟练技术工人。继续支持荆楚理工学院的建设和发展。加大人才开发力度，加强技能型和创新型人才的培养、引进和使用工作。全力抓好省十三运会筹备工作，开工建设钟祥体育馆、京山网球训练中心等一批体育基础设施。加快市博物馆筹建步伐。加强农村基层文化建设，开展丰富多彩的群众性文化体育活动。完善基本医疗和公共卫生服务体系，落实重大疾病防治措施，全面完成乡镇卫生院改扩建工作。推进“婚育新风进万家活动”国家示范市建设，完善计划生育利益导向机制，稳定低生育水平。完成第二次经济普查任务。继续做好“双拥共建”和优抚安置工作，加强国防动员和民兵预备役、人民防空建设。做好对口支援工作。积极推进民族、宗教、广播电视、新闻出版、文物保护、烟草专卖、妇女、儿童、老龄、残疾人、档案、地方志、外事、侨务、对台、仲裁、气象、防震减灾等社会事业发展。

　　保持社会和谐稳定。加强城乡基层自治组织建设，健全民主制度，发展基层民主，切实维护群众民主权利。深入开展“五五”普法活动，加强法制宣传教育，形成学法、用法、守法的良好社会氛围。广泛开展群众性精神文明创建活动，大力实施公民道德建设工程。加强网络管理，开展心理健康教育，积极营造文明健康的网络舆论氛围。深入推进平安荆门建设，加强社会治安综合治理，健全“群防群治”的社会治安防控体系，完善治安视频监控系统，依法打击各类犯罪活动。严格落实稳定工作责任制，畅通信访渠道，完善社会矛盾纠纷排查化解机制，加强人民调解工作机制建设，主动防范和妥善化解社会矛盾。加强公共安全和应急管理工作，完善突发事件应急管理体制和机制，提高处置突发事件能力。继续实施食品药品放心工程，强化对食品、药品和饮食卫生的监管。毫不放松地加强安全生产管理和监督，减少安全事故发生。

　　各位代表，今年市政府将继续围绕关系人民群众切身利益的问题，为人民群众办好10件实事。1.城镇新增就业4万人，组织农村劳动力转移培训5万人，通过政策扶持实现自主创业2500人，带动就业1万人。2.建设廉租住房2.3万平方米；对城市无房或人均住房建筑面积11平方米以下的低收入家庭发放廉租住房补贴。3.继续抓好中心城区粉尘、烟尘、废气等污染治理，中心城区环境空气质量达到国家二级标准以上的天数占90%以上；实施竹皮河后续整治和杨树港河道综合整治，建成南城区污水处理厂；改造20条小街小巷。4.解决21万人的农村饮水安全问题；新建农村户用沼气池4万口。5.新建通村公路460公里，新建通组公路碎石路面1000公里、路基2000公里；新建85个候车棚、150个招呼站。6.建设40个村级文化活动中心或文化中心户，建设120个农民体育健身工程项目；建成“农家书屋”200家；改造和建设400家农家店。7.加大扶贫助学力度，资助100名应届荆门籍大学贫困新生入学；对中等职业学校1.8万名城乡家庭经济困难的一、二年级学生进行资助；免费为1500名城市家庭经济困难学生提供教科书；免除市特殊教育学校所有在校学生学杂费、课本费和生活费。8.在南城区新建一所九年一贯制学校，以三甲标准建设一所综合性医院。9.实施社区“三基”行动计划（基础设施、基本队伍、基本工作规范），用两年左右时间分批基本解决中心城区社区的标准化建设问题。10.为困难群众和农民工提供法律援助，保障弱势群体的合法权益。

　　（八）加强政府自身建设，提高行政效能

　　面对新形势、新任务，我们必须不断增强忧患意识和责任意识，切实转变政府职能，提高行政效能，进一步增强政府执行力、公信力和约束力，努力打造人民群众满意的服务型政府。

　　坚持务实高效，提高执行力。深入开展学习实践科学发展观活动，进一步解放思想，鼓励创新，宽容失败，敢于突破，敢于担当，营造干事创业的浓厚氛围。坚持程序服从效率，权力服从效果，工作谋划注重长远，工作推动把握细节，工作方法力求创新，工作落实讲求实效，出色地承担起改革之责、发展之责、稳定之责。健全完善目标责任管理和绩效评价指标体系，强化过程管理和督促检查，切实解决“执行不够、落实不力”的问题，确保责任明确到位、政令执行到位、奖惩兑现到位、各项工作落实到位。

　　坚持秉公用权，提升公信力。强化制度信用、政策信用和程序信用，建立健全部门服务公开承诺制和岗位责任制，言必行、行必果，全面提升政府诚信。深入推进政府信息公开，加强电子政务建设，完善新闻发布制度，丰富与公众互动方式，切实保障人民群众的知情权、参与权、表达权、监督权。坚持民主决策，健全领导、专家、群众相结合的决策机制，落实决策咨询、听证、公示制度，确保决策反映民愿、体现民意、集中民智。始终秉持亲民爱民的民本情怀，切实解决好人民群众最关心、最直接、最现实的利益问题，把全市上下的智慧和力量凝聚到加快荆门崛起的大业上来。

　　坚持依法行政，增强约束力。自觉接受人大及其常委会的法律监督和工作监督，接受政协的民主监督以及社会舆论监督，认真办理人大议案、代表建议和政协提案。支持监察、审计部门依法独立开展工作，充分发挥各民主党派、工商联和无党派人士的民主监督作用。着力推进依法治市工作，加强市县政府依法行政，严格依照法定权限和程序行使权力、履行职责。完善行政执法责任制和行政复议制度，规范行政处罚自由裁量权，强化执法队伍教育培训，切实做到严格执法、公正执法、文明执法。牢固树立过“紧日子”思想，厉行节约、反对浪费，进一步精简会议和文件，努力降低行政成本。认真执行领导干部廉洁从政各项规定，严肃查处违法违纪案件，坚决刹住干部私驾公车、抹牌赌博和公款吃喝等歪风，真正做到以廉洁取信于民，以勤政造福于民。

　　各位代表！危机孕育希望，奋斗铸就伟业。任何困难都改变不了荆门人民建设美好家园的强烈愿望，任何挑战都阻挡不了荆门人民加快开放崛起的坚定步伐。让我们紧密团结在以胡锦涛同志为总书记的党中央周围，在省委、省政府和市委的坚强领导下，全面落实科学发展观，充分调动一切积极因素和发展力量，上下一心，和衷共济，提振信心，共克时艰，为加快荆门崛起、实现全面小康而努力奋斗！
